# Detection of needle tract implantation and peritoneal seeding after radiofrequency ablation using intraoperative near-infrared fluorescence system for recurrent hepatocellular carcinoma: a case report

**DOI:** 10.1186/s40792-018-0485-5

**Published:** 2018-07-13

**Authors:** Masashi Nakamura, Shinya Hayami, Masaki Ueno, Manabu Kawai, Atsushi Miyamoto, Norihiko Suzaki, Seiko Hirono, Ken-ichi Okada, Motoki Miyazawa, Yuji Kitahata, Hiroki Yamaue

**Affiliations:** Second Department of Surgery, School of Medicine, Wakayama Medical University, 811-1 Kimiidera, Wakayama, 641-8510 Japan

**Keywords:** Hepatocellular carcinoma (HCC), Radiofrequency ablation (RFA), Needle tract implantation, Peritoneal seeding, Indocyanine green (ICG), Near-infrared fluorescence (NIRF)

## Abstract

**Background:**

Radiofrequency ablation (RFA) for hepatocellular carcinoma (HCC) is already fully established worldwide. Needle tract implantation and peritoneal seeding occasionally occur by RFA, and the prognosis of these cases is thought to be poor. In this study, intraoperative real-time near-infrared fluorescence (NIRF) system by indocyanine green (ICG) incidentally detected both needle tract implantation and peritoneal seeding. As the utility of this system for identification of implanted and disseminated lesions after RFA for HCC has not been widely reported, we report a case of successful detection by real-time ICG-NIRF imaging and subsequent resection.

**Case presentation:**

A 76-year-old man originally underwent medial sectionectomy for HCC in 2009. When repeated intrahepatic recurrence occurred, he underwent RFA and transcatheter arterial chemoembolization (TACE) for recurrent HCC twice at segment III and once at segment IV. In 2013, the second hepatectomy for recurrent HCC at segment VIII was performed. In 2016, he had recurrent HCC at segment III around a previous RFA and TACE scar; therefore, left lateral sectionectomy was planned. ICG-NIRF system was used to observe a main intrahepatic metastasis at segment III and to search for other tumors in the remnant liver. Although there was no signal on the surface of the remnant liver, tiny signals were observed in the abdominal wall and greater omentum. These tumors were on the needle tract of the previous RFA; both lesions, therefore, were resected. These tumors were pathologically proven to be HCC metastases. The patient has had no recurrence 14 months after the last hepatectomy.

**Conclusions:**

ICG-NIRF system might be helpful in the detection of not only intrahepatic lesions but also needle tract implantations or peritoneal seeding. RFA should be avoided in patients with high risk of needle tract implantation and peritoneal seeding.

## Background

Radiofrequency ablation (RFA) is one curative treatment option for hepatocellular carcinoma (HCC). The safety and outcomes of RFA are comparable to those of surgical treatments for small HCC. Kudo et al., on the other hand, reported the frequency of major complications after RFA as being up to 8.9% [1–3]. Needle tract implantation and peritoneal seeding after percutaneous RFA for HCC was reported by Llovet et al. [4]. Frequency of needle track seeding was reported as 12.5% (4/32 cases). Dissemination after RFA might not, however, occur at such a high frequency and it is almost absent in many reports from Japan [2]. Tumor implantation and peritoneal seeding after percutaneous RFA are considered to be a highly advanced stage and the operative indication for implanted HCC might be limited [5].

Intraoperative detection of HCC using near-infrared fluorescence (NIRF) system was reported by Gotoh et al. and Ishizawa et al. [6, 7]. This technique uses indocyanine green (ICG) and has been intraoperatively applied to liver surgery to detect novel lesions for around a decade. The usefulness of NIRF system for the detection of needle track seeding after RFA is, however, not well reported.

In this study, implanted and disseminated lesions were incidentally detected and then resected in one case using NIRF system during an operation for recurrent HCC after RFA. We present a case of real-time ICG-NIRF system being used to detect implanted and disseminated lesions.

## Case presentation

**Fig. 1:**
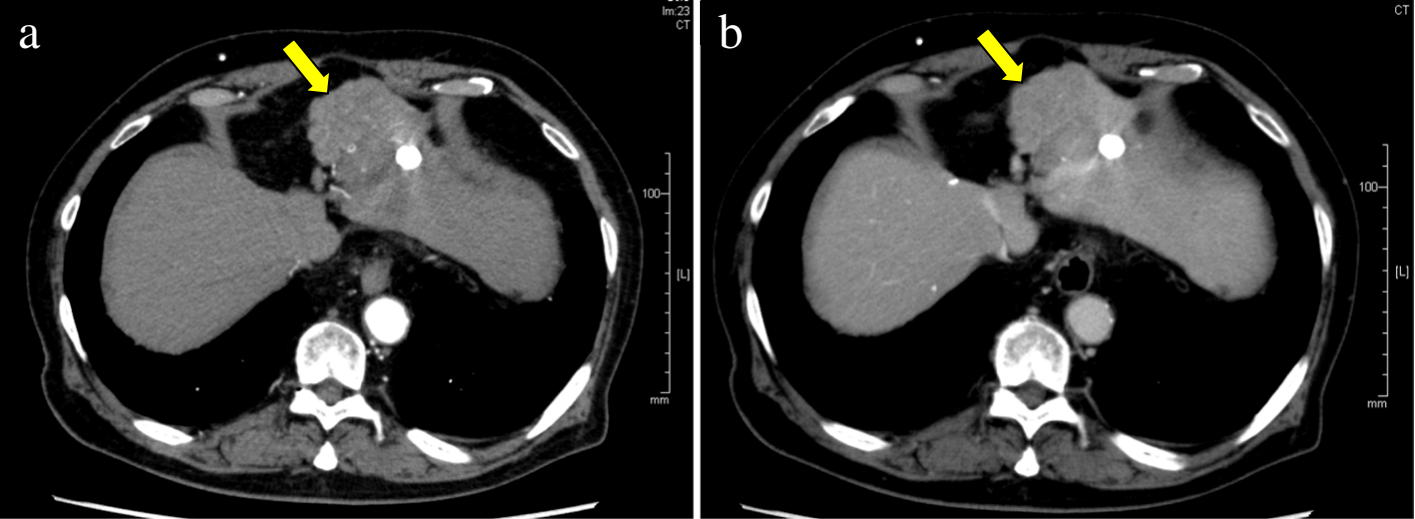
Enhanced computed tomography (CT) showed a recurrent lesion with radiofrequency ablation (RFA) and transcatheter arterial chemoembolization (TACE) scar inside (30 mm in diameter) at the surface of segment III. **a** Early arterial phase. **b** Delayed phase

A 76-year-old man originally underwent medial sectionectomy for HCC in 2009. When repeated intrahepatic recurrence occurred, he underwent RFA and transcatheter arterial chemoembolization (TACE) for recurrent HCC twice at segment III and once at segment IV. A 1.5-cm-diameter tumor at segment III was ablated by RFA needle twice for the first time. Four months after this treatment, a 1-cm diameter of new recurrence around the previous lesion at segment III was pointed out and ablated once by RFA needle. In 2013, the second hepatectomy for recurrent HCC at segment VIII was performed. In 2016, he had recurrent HCC at segment III around a previous RFA and TACE scar again; left lateral sectionectomy was therefore planned (Fig. 1).

The patient had persistent infection of chronic hepatitis C and diabetes requiring insulin. Laboratory data before this operation is shown in Table 1. Focused on tumor markers, des-gamma carboxyprothrombin (DCP) was high while alpha-fetoprotein (AFP) and lectin-reactive alpha-fetoprotein (AFP-L3%) were within normal limits. Liver function was good, Child-Pugh score 5A. We therefore considered these tumors to be within operative indication for left lateral sectionectomy as the third operation on the clinical diagnosis of recurrent HCC of Union for International Cancer Control (UICC) TNM staging stage IB (T1bN0M0).

**Table 1:** Laboratory data on admission

Value		Unit	Value		Unit
WBC	61.6	(10^2^/μl)	Alb	4.6	(g/dl)
RBC	425	(10^4^/μl)	AST	20	(IU/l)
Hb	12.7	(g/dl)	ALT	15	(IU/l)
Ht	38.2	(%)	ALP	283	(IU/l)
PLT	20.9	(10^4^/μl)	T-bil	0.6	(mg/dl)
Neu	70.2	(%)	D-bil	0.1	(mg/dl)
Eosin	6.8	(%)	Cre	1.48	(mg/dl)
Baso	0.6	(%)	eGFR	36.5	
Mono	5.5	(%)	BUN	26	(mg/dl)
Ly	16.9	(%)	Na	139	(mEq/l)
CRP	0.33	(mg/ml)	K	4.4	(mEq/l)
CEA	3.4	(ng/ml)	PT (ratio)	93	(%)
CA19-9	5.6	(UA/ml)	PT-INR	1.03	
AFP	1.9	(ng/ml)	ICG R_15_	8	(%)
AFP-L3%	Undetectable	(%)	HA	127	(ng/ml)
DCP	140	(mAU/ml)	HbA1c	8	(%)

*WBC* white blood cell, *RBC* red blood cell, *Hb* hemoglobin, *Ht* hematocrit, *PLT* platelet, *Neu* neutrophil, *Eo* eosinophil, *Baso* basophil, *Mono* monocyte, *Ly* lymphocyte, *CRP* C-reactive protein, *CEA* carcinoembryonic antigen, *CA19-9* carbohydrate antigen 19-9, *AFP* alpha-fetoprotein, *AFP-L3%* lectin-reactive alpha-fetoprotein, *DCP* des-gamma carboxyprothrombin, *Alb* albumin, *AST* aspartate aminotransferase, *ALT* alanine aminotransferase, *ALP* alkaline phosphatase, *T-bil* total bilirubin, *D-bil* direct bilirubin, *Cre* creatinine, *eGFR* estimated glomerular filtration rate, *BUN* blood urea nitrogen, *Na* natrium, *K* kalium, *PT* prothrombin time, *PT-INR* international normalized ratio of prothrombin time, *ICG R*_*15*_ indocyanine green excretion rate after 15 min, *HA* hyaluronic acid, *HbA1c* glycated hemoglobin

**Fig. 2:**
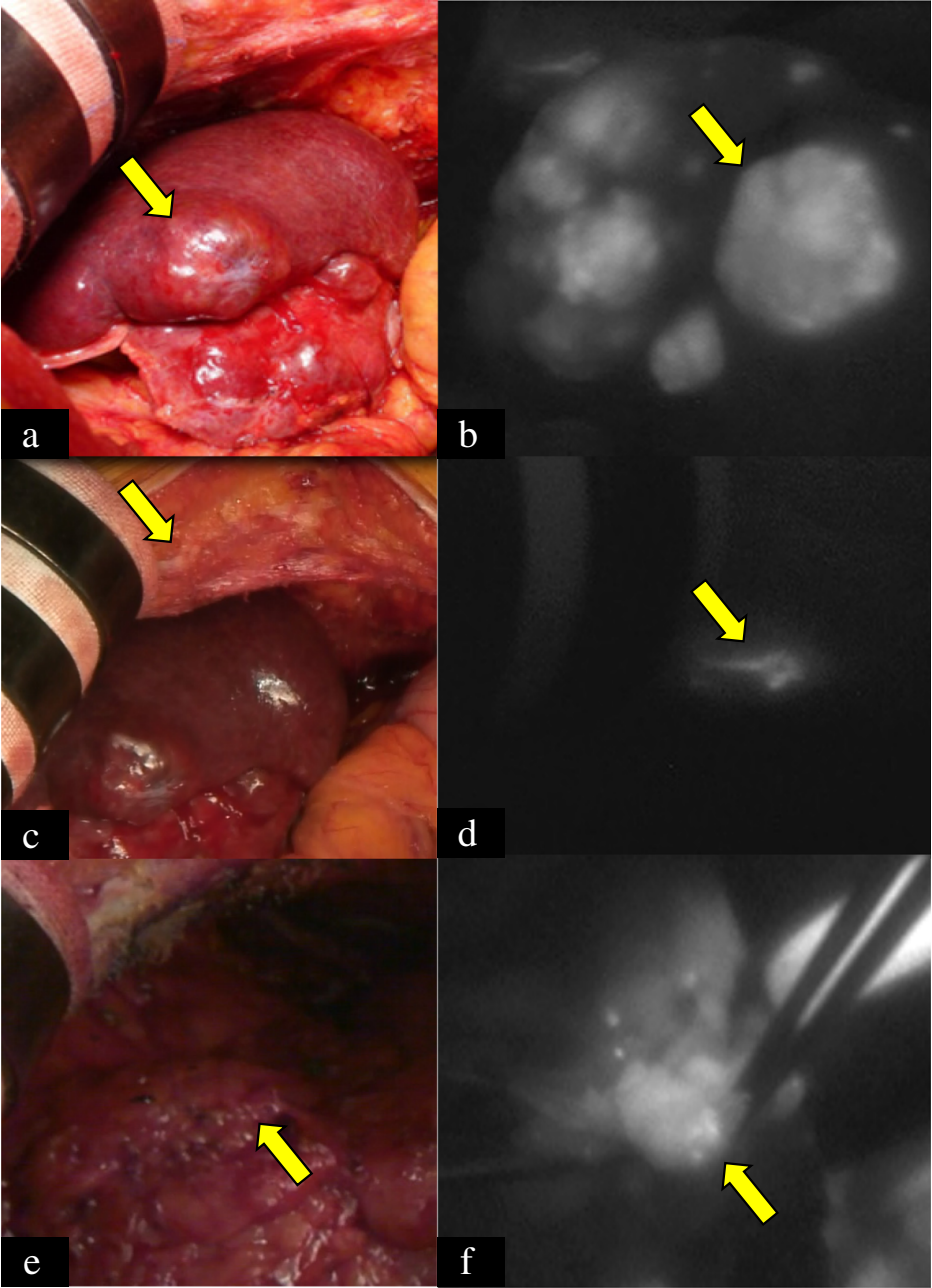
Intraoperative findings using near-infrared fluorescence (NIRF) system. **a** Recurrent lesions were located at the surface of segment III. **b** Fluorescent image of the tumors at segment III. **c** A lesion of the abdominal wall was detected. **d** Fluorescent image of the abdominal wall lesion. **e** A lesion of the abdominal wall was detected. **f** Fluorescent image of the lesion in the greater omentum

**Fig. 3:**
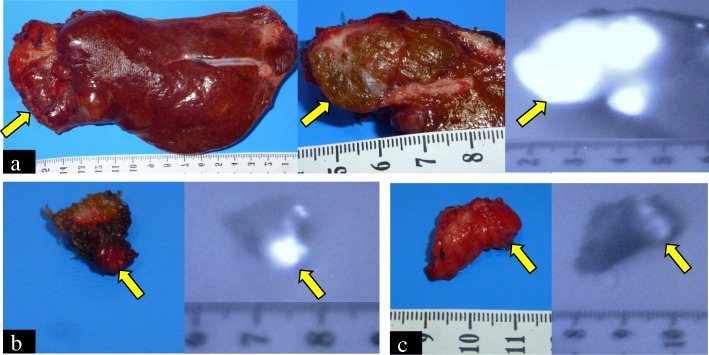
Specimen findings using near-infrared fluorescence (NIRF) system. **a** Tumors at segment III. **b** Abdominal wall. **c** Greater omentum

We planned to use ICG-NIRF system to observe the main intrahepatic metastasis at segment III and searched for other tumors in the remnant liver. Two days before the operation, 0.5 mg/kg ICG (Diagnogreen, Daiichi-Sankyo, Tokyo, Japan) was intravenously injected. Photodynamic eye (PDE, Hamamatsu Photonics, Hamamatsu, Japan) was used as a detector of NIRF. Intraoperative gross appearance is shown in Fig. 2a. The recurrent tumor was located at the liver surface of segment III by fluorescent signal that could be detected by NIRF system (Fig. 2b). There was no signal on the surface of future remnant liver (right lobe). We incidentally observed tiny signals in the right upper abdominal wall, however (Fig. 2c, d). Another fluorescent tumor was detected in the greater omentum (Fig. 2e, f). These tumors were thought to be seeding of the previous RFA, but localized and controlled. After left lateral sectionectomy, they were also resected. Fluorescent signals were observed from all tumors in each specimen, even after resection (Fig. 3). Operation time was 141 min and intraoperative bleeding was 230 ml.

**Fig. 4:**
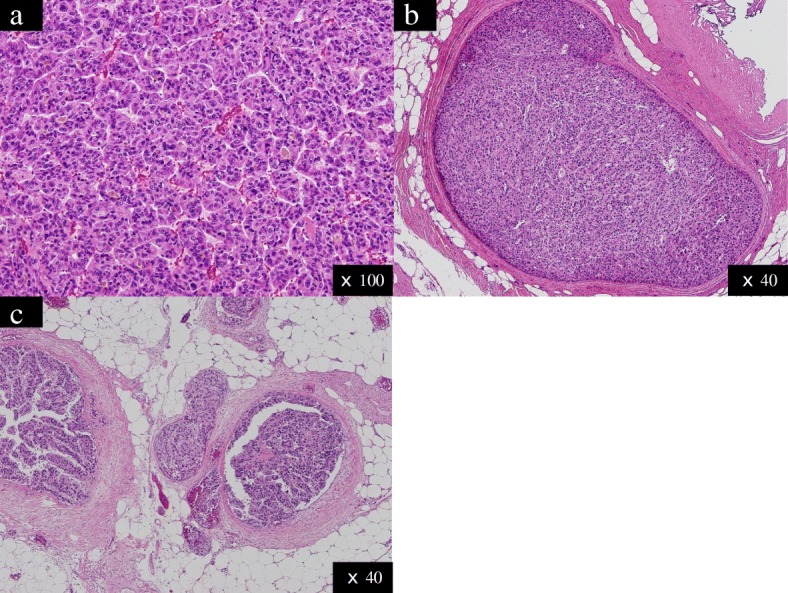
Pathological images of the resected specimens. **a** Tumors at segment III. **b** Abdominal wall. **c** Greater omentum

Histopathological examination revealed that the main recurrent liver tumor was moderately differentiated HCC showing the progression pattern of pseudoglandular type, including the bile duct with microvascular invasion for portal vein (vp1) (Fig. 4a). The main tumor included both viable and necrotic areas because of the previous TACE and RFA. Both abdominal wall and greater omentum lesions were pathologically demonstrated to be metastases of HCC (Fig. 4b, c).

The patient was discharged from our hospital on the eighth postoperative day without any complications. There has been no further recurrence 14 months after the operation.

### Discussion

In this study, we incidentally detected implanted and disseminated needle tract and peritoneal seeding from previous RFA by intraoperative NIRF imaging system. Detection of intrahepatic HCC was reported by Gotoh et al. [6] and Ishizawa et al. [7]. There are several reports about HCC detection using ICG-NIRF system [8, 9]. Moreover, this system could also be used to detect extrahepatic HCC lesions, such as in the appendix [10], lymph node, lungs, peritoneum, and adrenal glands [11]. The detailed mechanism of how extrahepatic tumor cells uptake ICG and emit fluorescence remains unclear. Satou et al. hypothesized that the extrahepatic HCC cells themselves also possess the capability to take up ICG from the blood stream. The absence of other adjacent cells or vessels, including the biliary system, may have caused the ICG retention in the metastatic lesions [11]. Further pathological examination is needed to understand this phenomenon.

This patient underwent repeated RFA for recurrent HCC. Percutaneous procedures have a risk of needle tract implantation, and peritoneal seeding and frequency of these complications are reported as between 0.9 and 12.5% [4, 12–14]. Llovet et al. indicated risk factors for complications include subcapsular lesions, use of cool-tip-type electrode needles, poorly differentiated HCC, and high levels of alpha-fetoprotein.

Efficacy of resection for needle tract implantation was suggested by Noda et al. [15]. One-year survival rate was 85% for patients who underwent resection of extrahepatic lesions, according to Satou et al. [11]. Operative indications are generally restricted to selective patients, however [5]. Time intervals between RFA and diagnosis of needle tract implantation were between 3 weeks and 48 months [16]; the mean doubling time was 112 (range 22–415) days [17]. ICG-NIRF system might be useful for the removal of these lesions during the operation for RFA treatment.

ICG-NIRF system has some limitations, however. The system is restricted to detection of fluorescence for tumors 5–10 mm from the liver surface [8, 18]. There were also false-positive results for liver cysts and dysplastic tumors in severe liver cirrhosis [8]. In the detection of extrahepatic lesions, however, there are no such limitations. This system might be helpful in the detection of needle tract implantations or peritoneal seeding.

## Conclusions

The current case study shows indication that ICG-NIRF system is helpful in the detection of needle tract implantations and peritoneal seeding as well as intrahepatic lesions.

## Abbreviations

HCC: Hepatocellular carcinoma
ICG: Indocyanine green
NIRF: Near-infrared fluorescence
RFA: Radiofrequency ablation
